# New generation geostationary satellite observations support seasonality in greenness of the Amazon evergreen forests

**DOI:** 10.1038/s41467-021-20994-y

**Published:** 2021-01-29

**Authors:** Hirofumi Hashimoto, Weile Wang, Jennifer L. Dungan, Shuang Li, Andrew R. Michaelis, Hideaki Takenaka, Atsushi Higuchi, Ranga B. Myneni, Ramakrishna R. Nemani

**Affiliations:** 1 Department of Applied Environmental Science, California State University – Monterey Bay, Seaside, CA USA; 2 NASA Ames Research Center, Moffett Field, CA USA; 3 Guizhou Provincial Key Laboratory of Geographic State Monitoring of Watershed, Guizhou Education University, Guiyang, China; 4 Bay Area Environmental Research Institute, Moffett Field, CA USA; 5 JAXA Earth Observation Research Center, Tsukuba, Ibaraki Japan; 6 Center for Environmental Remote Sensing, Chiba University, Chiba-shi, Chiba Japan; 7 Earth & Environment Department, Boston University, Boston, MA USA

**Keywords:** Ecological modelling, Carbon cycle, Forest ecology

## Abstract

Assessing the seasonal patterns of the Amazon rainforests has been difficult because of the paucity of ground observations and persistent cloud cover over these forests obscuring optical remote sensing observations. Here, we use data from a new generation of geostationary satellites that carry the Advanced Baseline Imager (ABI) to study the Amazon canopy. ABI is similar to the widely used polar orbiting sensor, the Moderate Resolution Imaging Spectroradiometer (MODIS), but provides observations every 10–15 min. Our analysis of NDVI data collected over the Amazon during 2018–19 shows that ABI provides 21–35 times more cloud-free observations in a month than MODIS. The analyses show statistically significant changes in seasonality over 85% of Amazon forest pixels, an area about three times greater than previously reported using MODIS data. Though additional work is needed in converting the observed changes in seasonality into meaningful changes in canopy dynamics, our results highlight the potential of the new generation geostationary satellites to help us better understand tropical ecosystems, which has been a challenge with only polar orbiting satellites.

## Abstract (web summary)

Cloud cover and scarcity of ground-based validation hinder remote sensing of forest dynamics in the Amazon basin. Here, the authors analyse imagery from a high-frequency geostationary satellite sensor to study monthly NDVI patterns in the Amazon forest, finding support for spatially extensive seasonality.

## Introduction

Amazon forests have a strong influence on global climate, carbon, and water cycles. Understanding the interactions of Amazon forests with the climate system is a key to predicting climate changes in the 21st century^1,2^. For instance, severe Amazon droughts are expected to occur more frequently as a result of global warming^3,4^ and modeling forests’ responses to drought is crucial for climate projections^5^. Despite the importance of Amazon vegetation dynamics in climate change studies, the lack of ground observations still leaves large uncertainties in how to represent tropical evergreen forests in Earth system models^6^. While efforts to monitor Amazon forests in situ continue,^7,8^ optical remote sensing plays a major role in monitoring vegetation dynamics for broad regions in the Amazon.

Historically, long-term records of Normalized Difference Vegetation Index (NDVI) have been the most useful data source among various optical remote sensing datasets derived from sun-synchronous sensors such as the Advanced Very High Resolution Radiometer (AVHRR) and the Moderate Resolution Imaging Spectroradiometer (MODIS)^9^. In theory, AVHRR and MODIS can observe most places on the Earth at least once a day during daytime. However, in tropical regions, land surface monitoring by optical remote sensors is very often obstructed by clouds. In particular, the Amazon basin experiences over 2000 mm/year of precipitation^10^ and cloud obscuration occurs throughout the year. Therefore, in practice AVHRR and MODIS cannot provide sufficient clear-sky observations over Amazon forests to generate robust statistics that summarize the vegetation dynamics. This issue has contributed to inconsistent conclusions among various Amazon forest studies, even though they all tend to be based on the same satellite datasets. For instance, studies on Amazon forest seasonality based on Vegetation Indices (VI) disagree on whether there is more greenness in the dry season than in the wet season^11–15^. The impact of droughts on Amazon forests has also been debated^16–19^. A reported decadal decline of greenness in the Amazon forest canopy may or may not be caused by drought^20,21^. Conflicting conclusions among these studies arise from different interpretations of VI signals, which depend on algorithms that handle cloud contamination^22^. The state-of-the-art cloud masking techniques in the Multi-Angle Implementation of Atmospheric Correction algorithm (MAIAC)^23^ are reported to increase clear-sky observations two to five times compared to traditional methods^24^, but clear-sky observations still may not be available within critical composite periods. Therefore, the NDVI data derived from existing optical remote sensing sensors alone are insufficient to end controversies regarding Amazon vegetation dynamics due to aerosol, water vapor, and cloud contamination^25^.

In this study, we examine the Amazon basin cloud contamination issue using data from the new Advanced Baseline Imager (ABI) onboard the Geostationary Operational Environmental Satellite 16 (GOES-16) satellite at 1-km resolution and compare these measurements to tower-based observations of monthly mean Net Ecosystem Exchange (NEE) or CO_2_ flux at the top of the canopy at two sites - see Methods. GOES-16 was launched in November 2016. It carries ABI with a high-frequency observing capability and 16 spectral bands, which include a red band (band 2) and a near infrared band (band 3), similar to the corresponding bands of AVHRR or MODIS^25^. ABI’s full disk scan covers the entire Amazon basin. In 2018, the ABI default mode of scanning was Mode 3, which performs full disk scans every 15 min. The default mode of ABI was changed to Mode 6 in 2019, which provides full scan images every 10-minutes. No such high-frequency (and high-resolution) optical sensors exist for monitoring the entire Amazon basin from space before the advent of ABI.

A second objective of this paper is to use the GOES16 ABI data to assess seasonality in the greenness of Amazon evergreen forests. The details of seasonality in the leaf area index (LAI) of Amazon evergreen forests has been crucial for modeling carbon or water cycles in general circulation models. Due to the difficulty in estimating LAI at stand level from optical satellite imaging systems operating in visible – shortwave infrared (400–2500 nm) regions, long-term observations of LAI are limited in the Amazon basin (e.g. Ref. ^26^). Dozens of ground observations of monthly litterfall in the Amazon basin have shown a mild seasonality in litterfall^27^. However, the locations of those observations are biased near the edges of the Amazon basin, and the determining factor of litterfall seasonality is still unknown^27^. Therefore, remote sensing is necessary to help us understand the spatial patterns of seasonality in LAI of the Amazon evergreen forests. Vegetation Indices (VI) have been frequently used as surrogates for LAI to examine Amazon seasonality^11,12,28^. For example, increases in Enhanced Vegetation Index (EVI) have been observed in the dry season in the west Amazon basin^11^, but conflicting explanations for this phenomenon have been offered. The EVI increases were explained by an LAI increase^28^, an artifact of sun-sensor-geometry^12^, and changes in leaf age through the leaf flush^29^. These studies suffered from the lack of sufficient data due to cloud contamination^22^ hindering a fuller assessment of the capacity to estimate canopy biophysical parameters. In this study, instead of directly analyzing the EVI increase in the dry season, we provide evidence for the existence of seasonality in Amazon evergreen forests using frequent greenness (NDVI) observations from ABI. The interpretation of the physical meaning of NDVI is much easier than EVI due to NDVI’s simple formula, and the relationship with vegetation physical variables has been well established^30^. Furthermore, EVI has the disadvantage of a high sensitivity to sun-sensor-geometry^12,31^. Therefore, results derived from ABI NDVI are likely to be more robust than those from EVI to study changes in the greenness of the Amazon forest canopy^31^.

## Results

### 15-minute ABI NDVI in dry- and wet-season months

**Fig. 1:**
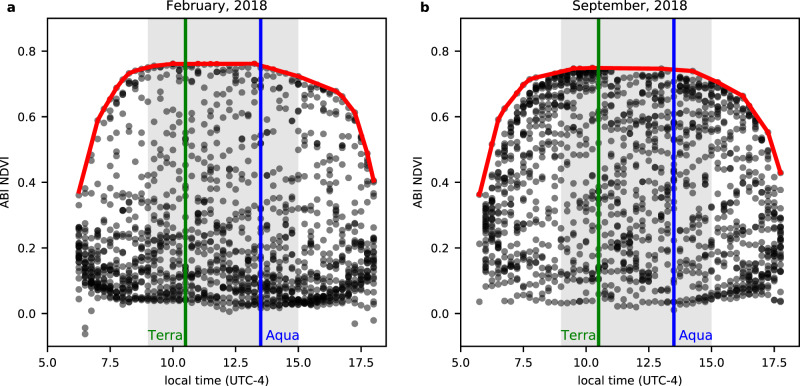
Scatterplot of Advanced Baseline Imager (ABI) Normalized Difference Vegetation Index (NDVI) against local time (UTC-4) at Km34 near Manaus. **a** February, 2018 (wet season). **b** September, 2018 (dry season). Each gray dot represents a 15-minute ABI NDVI value. The green line and blue lines show approximate overpass time for Terra and Aqua, respectively. The red lines are the convex hulls of the ABI NDVI scatterplots. The gray shaded areas indicate the 9 a.m. to 3 p.m. time window.

Monthly scatterplots of ABI NDVI against local time from a 1 km × 1 km pixel at Km34 (2.61°S, 60.21°W, Reserva Cuieiras)^32^ formed characteristic shapes in both wet season and dry season months (Fig. 1). Similar shapes exist in monthly plots throughout the year. Due to cloud contamination, there were more low values of NDVI in the wet season (Fig. 1a, 69% of NDVI is less than 0.3) than in the dry season (Fig. 1b, 41% of NDVI is less than 0.3). Using the maximum-value composite (MVC) logic which was originally developed to represent clear-sky NDVI of a specific duration from daily, often cloud-contaminated NDVI^33^, we assumed that the maximum NDVI value for each 15-minute interval in a month represents the clearest sky NDVI and is therefore representative of the vegetation canopy. Using this assumption, the clear-sky NDVI values are in the convex hull of the scatterplot (Fig. 1). In studies of solar-geometry effects on NDVI at the surface, higher NDVI is often observed in the early morning and late evening over forest targets. This is caused by relatively higher absorption of red light by the canopy at lower sun angles^34,35^. In contrast, the fall off of the Top-of-Atmosphere (TOA) NDVI in the morning and evening can be explained by the longer path-length of light through the aerosol-laden atmosphere in the early morning and the late evening^36^.

Since there were plateaus in the upper convex hull of NDVI from 9 a.m. to 3 p.m., we created daily MVCs over this 6-hour period to represent the daily clearest NDVI. There are 24 (6 h × 4 times per hour) chances of clear-sky conditions from ABI 15-minute data per day compared to two chances for MODIS (from Terra and Aqua). Therefore, the GOES platform improves chances of clear-sky observation much more than those obtained by any MODIS cloud-detection algorithm improvement^24^.

### Comparison of seasonal time-series between ABI and MODIS

**Fig. 2:**
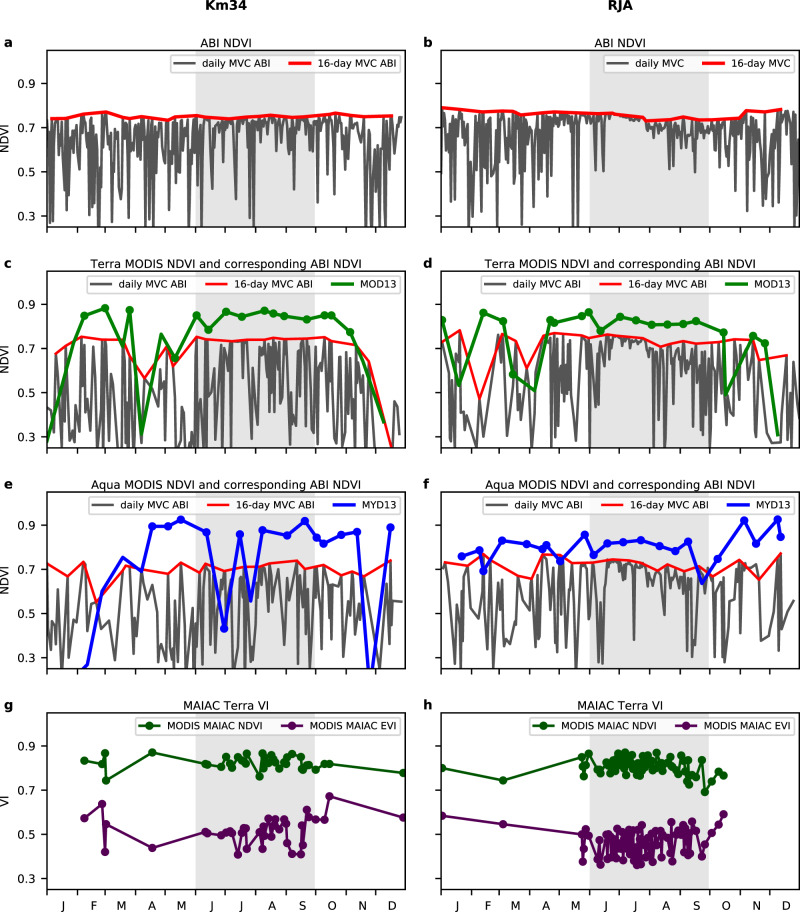
Time series of vegetation indices at two study sites in 2018. Left column is Reserva Cuieiras near Manaus (Km34, 2.61°S, 60.21°W) and right column is Reserva Jaru (RJA, 10.08°S, 61.93°W). **a**, **b** Time series of daily (black lines) and 16-day (red lines) Advanced Baseline Imager (ABI) Maximum Value Composite (MVC) Normalized Difference Vegetation Index (NDVI), where maximum NDVI values are composited over the time window from 9 a.m. to 3 p.m. (local time). **c**, **d** Daily (black lines) and 16-day (red lines) maximum NDVI values composited over the time window around 10:30 a.m., the approximate Terra overpass time. The green lines indicate time series of the corresponding Terra Moderate Resolution Imaging Spectroradiometer (MODIS) 16-day NDVI (MOD13A2). Data plotted with green dots had a reliability flag of good or useful in MODIS QA. **e**, **f** Daily (black lines) and 16-day (red lines) maximum NDVI values composited over the time window around 1:30 p.m., the approximate Aqua overpass time. The blue lines indicate time series of the corresponding Aqua MODIS 16-day NDVI (MYD13A2). Data plotted with blue dots had a reliability flag of good or useful in MODIS QA. **g**, **h** Daily NDVI (green lines) and Enhanced Vegetation Index (EVI) (purple lines) calculated from Terra Multi-Angle Implementation of Atmospheric Correction algorithm (MAIAC) daily reflectance. The dry season from June to September is shaded in gray in all plots.

We applied the daily MVC for 15-minute TOA NDVI from 9 a.m. to 3 p.m. to create the daily clearest sky NDVI at Km34 and RJA (10.08°S, 61.93°W, Reserva Jaru)^37^ in 2018. To remove the fluctuations of the daily MVC NDVI due to residual cloud contamination, we also created the 16-day MVC NDVI using the same composite period as the MOD13A2 NDVI product. At Km34, the 16-day MVC NDVI shows far smaller fluctuations than the daily MVC NDVI, and we did not observe any seasonality in the 16-day MVC NDVI. The 16-day MVC NDVI has the same NDVI magnitude in the wet season as in the dry season (Fig. 2a). In contrast, the 16-day MVC NDVI at RJA had a low NDVI (0.74) from August to November in the late dry season compared to a high NDVI (0.78) in the other months (Fig. 2b).

We simulated the daily MODIS NDVI by extracting ABI NDVI at the approximate MODIS overpass time (10:30 a.m. for Terra and 1:30 p.m. for Aqua) and created 16-day MVC NDVI from the daily ABI NDVI for both Terra and Aqua overpass times. We compared them with the time series of the standard MODIS NDVI products for Terra (Fig. 2c, d) and Aqua (Fig. 2e, f). Even though MODIS NDVI was calculated from surface reflectance and had a different compositing scheme (Fig. 2c–f), the seasonal variation of MODIS NDVI is similar to that of the simulated ABI TOA NDVI, especially in Fig. 2c. Both ABI and MODIS NDVI fluctuated more than 0.1, and large drops (from 0.75 to 0.6) happened in the wet season. Those large drops (from 0.75 to 0.5) were much higher than those in the 16-day MVC ABI in the dry season at RJA (Fig. 2b). These drops indicated that 16-day MVC from once-a-day observations could not remove cloud contamination in the wet season. The MODIS QA reliability flag helped to pick up clear-sky pixels in the dry season, but often flagged the cloud-contaminated pixels as good or useful data in the wet season. At RJA, both Terra (MOD13A2) and Aqua (MYD13A2) MODIS NDVI showed a slight decrease from August (0.8) to November (0.75) similarly to the 16-day MVC ABI NDVI; however, those small decreases were indiscernible due to the frequent cloud drops, which hid the dry season signals.

MAIAC found more clear-sky observations from MODIS data than in MOD13A2 and MYD13A2 data using its sophisticated cloud detection algorithm (Fig. 2g, h). However, the clear-sky observations found by MAIAC were mostly in the dry season, and the number of clear-sky data in the wet season was only slightly larger than that from MOD13A2 and MOYD13A2 data. MAIAC could not find any clear-sky days in some months, for example November at both Km34 and RJA. Therefore, the number of clear-sky observations in the MODIS MAIAC product was still insufficient in the wet season to detect seasonality of Amazon evergreen forests.

At Km34, the MAIAC NDVI data showed no seasonality in NDVI, while MAIAC EVI increased from July to October (Fig. 2g) as reported in several studies^11,12,15,26,29,38^. Meanwhile, the MAIAC EVI at RJA steeply increased in October (from 0.4 to 0.7), but MAIAC NDVI showed no seasonal trend (Fig. 2h). The period of lower MAIAC EVI at RJA occurred much earlier from June to August than did 16-day MVC ABI NDVI.

These findings indicate that the detected seasonality in the Amazon forest greenness from a sun-synchronous satellite sensor can be spurious and often caused by cloud contamination even using a state-of-the-art cloud screening algorithm. The influence of cloud contamination on the MODIS VIs is so strong that even monthly composited MODIS VI data are not suitable for seasonality analyses (Fig. 2c–h). The high-frequency observations from ABI can reduce the effect of chronic cloud contamination in the Amazon basin and allow the study of tropical forest seasonality.

### Number of clear-sky observations

**Fig. 3:**
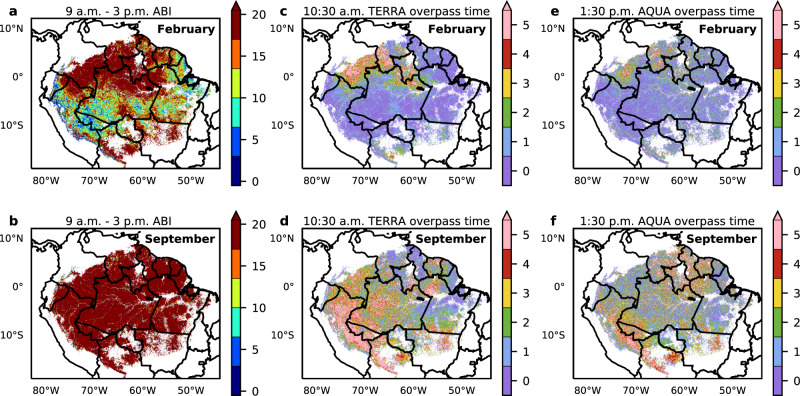
Number of clear-sky observations per month by Advanced Baseline Imager (ABI) in wet and dry seasons in 2018. Top row is February, 2018 (the wet season) and bottom row is September, 2018 (the dry season). In particular, **a** and **b** show the numbers for the time window from 9 a.m. to 3 p.m. (local time). **c**, **d** The numbers for the time window at 10:30 a.m. **e**, **f** The corresponding numbers for a time window at 1:30 p.m.

**Fig. 4:**
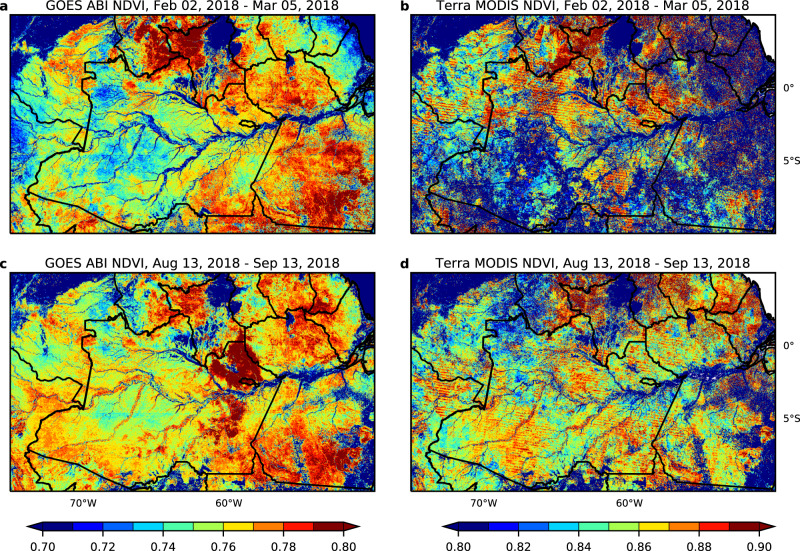
Comparison between 2018 Advanced Baseline Imager (ABI) and Moderate Resolution Imaging Spectroradiometer (MODIS) monthly Maximum Value Composite (MVC) Normalized Difference Vegetation Index (NDVI). **a** and **b** are NDVI composites from February 2nd to March 5^th^ (wet month). **c** and **d** are NDVI composites from August 13^th^ to September 13^th^ (dry month). **a** and **c** are GOES-16 ABI composites; **b** and **d** are Terra MODIS composites.

We counted the number of clear-sky observations in February (i.e., the wet season) from 9 a.m. to 3 p.m. in Amazon evergreen forests with Terra overpass time at 10:30 a.m. and Aqua overpass time at 1:30 p.m. (Fig. 3 and Table 1). Using the results from Fig. 1, the count of clear-sky observations was defined as the number of NDVI values that are within 0.03 of the convex hull of NDVI between 9 a.m. and 3 p.m. In the wet season, at least five clear-sky observations of ABI were available from any pixel in the entire Amazon evergreen forest zone. On average, ABI provided 23.6 clear-sky observations in February, and 58.7 clear-sky observations in September for all pixels in the Amazon basin. When we restricted the observations to Terra or Aqua overpass times, ABI provided only one clear-sky opportunity at each time during the month of February. Observations in September were more numerous than in February, but the mean counts are still 2.5 or 2.1 at Terra or Aqua overpass time, respectively. Therefore, even a monthly composite of MODIS data failed to find the clear-sky conditions within a month, so that the analysis derived from MODIS data cannot be conclusive about Amazon evergreen forest seasonality. Since 2018 was a normal precipitation year^39^, the availability of clear days in wet years would likely be lower. In comparison, ABI had approximately 24 (ranging between 21–35 on a monthly basis) times more clear-sky observations than MODIS per year, which corresponds to the number of ABI observations per day from 9 a.m. to 3 p.m. (Supplementary Table 1). The frequency of ABI full-disk observations became 10 min after April 2019, increasing the potential of ABI for clear-sky observations. As a result, the map of monthly MODIS NDVI in the wet-season composite is inconsistent with the dry season map, while GOES ABI monthly composite maps are consistent through the year (Fig. 4).

**Table 1:** Mean number of clear-sky observations per month.

	February, 2018	September, 2018
9 a.m.–3 p.m. every 15 min	23.6	58.7
10:30 a.m. (Terra MODIS overpass time)	1.0	2.5
1:30 p.m. (Aqua MODIS overpass time)	0.9	2.1

The numbers of clear-sky observations per month (Fig. 3) are averaged over all of the Amazon evergreen forest zone for February, 2018 (the wet season) and September, 2018 (the dry season).

### Aerosol screening by MVC

The MVC can also screen out observations with high aerosol loading in addition to cloud contamination. Aerosols have the effect of decreasing NDVI, so MVC selects observations with low aerosol concentration^33^. During the dry season, aerosol concentrations observed in the Amazon basin can have high variability at hour-to-hour time scales^40^. The high-frequency observations of ABI thus also contribute to finding fewer aerosol-contaminated NDVI values than MODIS.

**Fig. 5:**
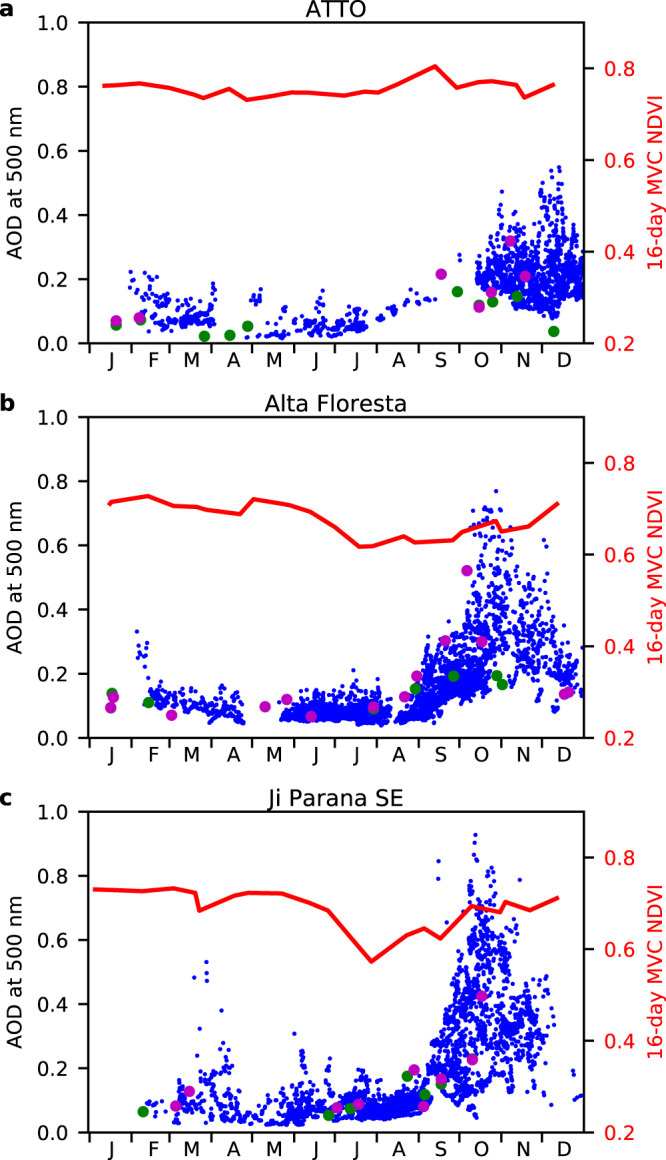
Time series of Aerosol Optical Depth (AOD) and 16-day Maximum Value Composite (MVC) Normalized Difference Vegetation Index (NDVI) at Aerosol Robotic Network (AERONET) sites in 2018. The blue dots show observed AOD at **a** Amazon Tall Tower Observatory (ATTO), **b** Alta Floresta, and **c** Ji Paramá SE. The magenta and green dots are AOD at overpass times of the MOD13A2 composite and 16-day ABI MVC NDVI, respectively. The red lines are 16-day MVC ABI NDVI.

To test the hypothesis that maximum-value compositing selects for observations with low aerosol concentrations, we extracted concurrent Aerosol Optical Depth (AOD) data corresponding to the 16-day MVC ABI NDVI and MODIS NDVI at three AERONET sites (Fig. 5) for 2018. The 15-min AOD data showed strong seasonality with high values from August to November, when cloud cover tends to be low. Aerosols from biomass burning hindered the observation of Amazon forest seasonality by MODIS^22^. The MODIS composite scheme tends to choose small values of AOD, but the chosen AOD was still over 0.5 (Fig. 5b). In comparison, the 16-day MVC of ABI occurred at times when the AOD is less than 0.3 for all the three sites. These results indicate that high-frequency ABI observations can help screen out high aerosol conditions.

NDVI seasonality is apparent at Floresta and Ji Parana SE (Fig. 5b, c). In particular, the low values of NDVI occurred before the rise of aerosol loading. Therefore, the timing of low NDVI was likely not caused by aerosol seasonality. The AOD differences between the biomass burning months and other months were 0.108, 0.058, and 0.104 at the Amazon Tall Tower Observatory (ATTO), Alta Floresta, and Ji Parana SE, respectively. A previous modeling study of NDVI sensitivity to AOD showed a linear decrease of 0.2 NDVI per increase of 1 AOD for high LAI vegetation^41^. Thus, the NDVI may decrease up to 0.022 (0.108 × 0.2) by the aerosol from biomass burning. To account for the aerosol effect, we set the threshold of NDVI detection as 0.022. If the NDVI seasonal amplitude is less than this threshold value, we suggest the NDVI variation is within the uncertainty of aerosol seasonality and cannot be ascribed to canopy foliage seasonality.

### Detecting statistically significant seasonal changes in evergreen forest greenness

**Fig. 6:**
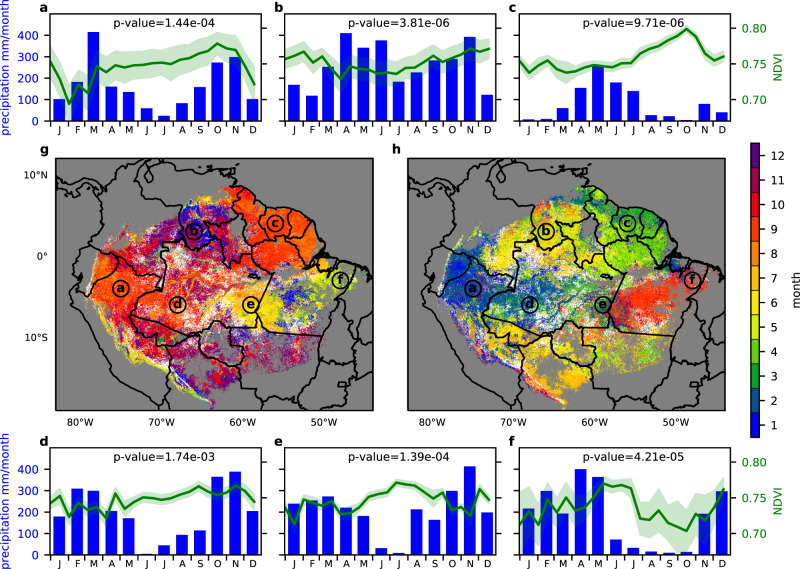
Normalized Difference Vegetation Index (NDVI) seasonality over Amazon evergreen forests in 2018. Panels **a** to **f** show example time series of 16-day Maximum Value Composite (MVC) NDVI corresponding to the six locations (**a**–**f**) indicated on the map **g** and **h**. The green line and envelopes are the mean and standard deviation, respectively, of NDVI taken from the 11 × 11 pixel region surrounding each location. The blue bars show monthly precipitation at each location. The two maps show the first month of the consecutive three months in which daily MVC NDVI reaches its (**g**) highest and (**h**) lowest values during 2018. Pixels where the high-to-low NDVI differences are statistically insignificant (*p* > 0.05) by the one-sided two-sample *t*-tests (*n* = 6) are masked out and shown in white.

To meet the second objective, we identified all the pixels in the Amazon evergreen forest zone where seasonality in remotely sensed greenness is detected. For each pixel, we calculated when the high-NDVI season and the low-NDVI season were observed (see Methods for details). Pixels with differences between low and high NDVI values of less than the chosen threshold (0.022) were treated as no difference due to the aerosol contamination uncertainty as discussed above. GOES ABI found a statistically significant difference in NDVI values, i.e., seasonality in NDVI, in 85% of the evergreen forest pixels (Fig. 6g, h). Both the high-NDVI season (Fig. 6g) and low-NDVI season (Fig. 6h) were spatially heterogeneous across the Amazon region. These regional differences were recognizable from the time-series plots of daily MVC ABI NDVI (Fig. 6a–f). For example, the southern Amazon forest showed a gradient of the greening timing from west (September: Fig. 6d) to east (May: Fig. 6f). The heterogeneous seasonality of Amazon evergreen forests appear to corroborate litterfall observations^27^.

The regional dependency of seasonality timing indicates that we cannot conclude the existence of seasonality in Amazon forests from a few intensive study sites, such as Manaus and Santarem. For instance, Fig. 2a showed no seasonality in NDVI at Manaus, but the surrounding area showed a significantly high-NDVI season from June to October (Fig. 6g, h). Additionally, some studies assumed that the growing season in Amazon is June to October deduced from the intensive observation sites, but the same seasonality was observed only in the southeastern part of Amazonas State and the western part of Pará State. To understand seasonal variation, the growing season should be defined from region to region.

The GOES ABI provides clearer evidence than MODIS to assess the existence of seasonality in the Amazon, and further to identify the timing of greening or browning. Previous carbon-flux studies have concluded that there is seasonality in photosynthesis in the Amazon region and that photosynthesis is higher in the dry season^8,42,43^. However, the discussion of seasonality of greenness or LAI has been confounded by different interpretations of MODIS EVI change^12,15,26^ and cloud contamination^22^. Our analysis confirms the existence of seasonality of greenness in 85% of the Amazon evergreen forest zone using the simple NDVI. Though there still remain issues inherent with optical remote sensing (see below in Discussion), our analysis indicates the area of forest exhibiting seasonality is about three times greater than previously reported using MODIS^13^. These results confirm the conclusion that LAI of Amazon forest has seasonality^28,44^ and shows that the seasonality of greenness exists in a much broader region and has a strong geographic dependency.

The response of NDVI to precipitation is also spatially heterogeneous and cannot be explained by simple biological mechanisms. As observed precipitation decreased in the dry season, NDVI increased in the south and west Amazon (Fig. 6a, d, e). Such responses were explained by changing responses to solar radiation^45^. However, the responses are different between high and low precipitation areas (Fig. 6b, c, f). The area with strong seasonality can be explained by a response to precipitation (Fig. 6c, f). However, the response of the higher precipitation area at Fig. 6b cannot be explained by a single climate driver, i.e., precipitation or solar radiation.

**Fig. 7:**
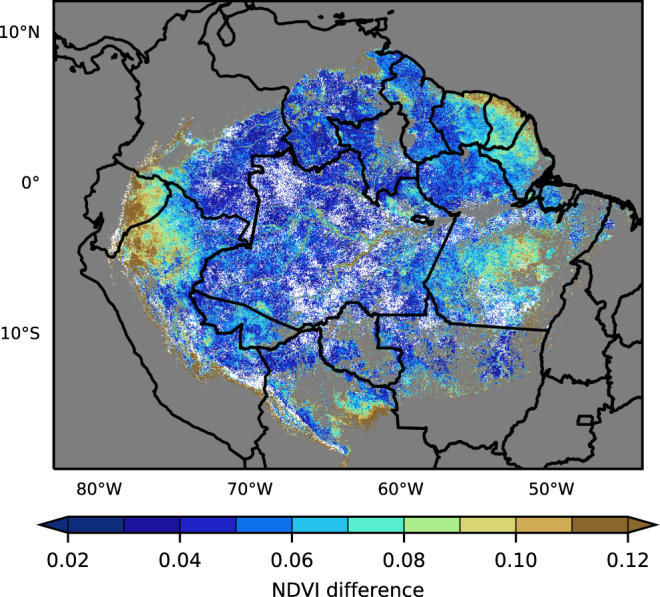
Map of the mean NDVI difference between the highest Normalized Difference Vegetation Index (NDVI) season and the lowest-NDVI season for each pixel over the evergreen forest in 2018. The white region had no statistically significant (*p* < 0.05) difference between the highest NDVI season and the lowest NDVI season by the one-sided two-sample *t* tests (*n* = 6).

Besides detecting the timing of seasonality, the ABI data also enabled us to examine NDVI differences in a more accurate manner. As seen in Fig. 2a, b, greenness differences are much smaller than fluctuations due to cloud contamination. The mean difference of NDVI between the highest value in the high-NDVI season and the lowest value in the low-NDVI season in 2018 was 0.07 for the evergreen forest (Fig. 7). The center of the Amazon basin showed a smaller NDVI difference which was less than 0.03 or statistically no difference. The Peruvian Amazon and French Guyana had a large NDVI difference of more than 0.1.

## Discussion

The GOES ABI data unveiled more detailed region-dependent seasonal changes in the 16-day MVC NDVI of Amazon evergreen forests than do MODIS data (Figs. 6 and 7). Although understanding the seasonality of gross primary production (GPP) of Amazon evergreen forests has been advanced with new sun-induced chlorophyll fluorescence (SIF) observations^43^ and flux modeling^42^, the seasonality in greenness (e.g. LAI and fraction of photosynthetically active radiation (FPAR)) has not been clearly revealed due to the small signal of the evergreen forest. The seasonality in greenness of Amazon evergreen forests has been studied using MODIS and the Geosciences Laser Altimeter System (GLAS) on the Ice, Cloud and land Elevation Satellite (ICESat). The EVI was originally introduced to detect small changes in high LAI values, but a lack of consensus on the interpretation of EVI change subsequently emerged with the increase in dry season EVI being attributed to cloud contamination, sun-sensor geometry, or the flushing of new leaves^46^. GLAS was designed for observing the atmosphere and ice sheets, but has also been used for estimating LAI. Unlike optical remote sensing, GLAS can avoid the cloud contamination issue, however, the small footprint and low temporal frequency observations by GLAS limit analyses to long time steps (a few months) and low spatial resolution (one-degree grid)^12,47^. As a result, GLAS barely shows LAI seasonality between the North Amazon and the South Amazon with uncertain month of start and end times^47^.

Though we showed that GOES ABI high-frequency observations improve cloud and aerosol screening compared to MODIS, residual atmospheric effects, such as sub-pixel cloud, still exist. To show the reproducibility of our results regardless of the residual atmospheric effects, we repeated the analysis for 2019 GOES ABI data and created the same figure as in Fig. 6 (Supplementary Fig. 2). In April 2019, the GOES ABI increased its observation frequency by changing scan mode, and scans the full disk every 10 min. Even though the frequency difference may have some influence on NDVI time series, the spatial patterns of the high-NDVI season and the low-NDVI season were comparable with 2018 (Fig. 6g, h and Supplementary Fig. 2g, h). More pixels with statistically insignificant differences were located in the center of the Amazon basin compared to 2018. This indicated that residual cloud effects still remained and obscured small greenness changes.

High-frequency ABI observations enable us to confirm the seasonality of Amazon evergreen forests by peeking at the land surface through the rarely occurring clear sky. The NDVI from sun-synchronous satellites cannot lead to effective analyses due to the impossibility of sufficient cloud screening. Thus, results from sun-synchronous satellites in this area have always been accompanied by doubt and suspected spurious conclusions. We showed that the seasonal amplitude of MODIS NDVI was magnified by cloud contamination. Long-term trend analysis using sun-synchronous satellites in perpetually cloudy areas is therefore also suspect. We strongly recommend the usage of GOES ABI to further the examination of trends in vegetation over the Amazon basin.

The eco-hydrological implications of the seasonal changes observed in satellite data have been studied extensively^48–51^. Generally, the NDVI is indicative of chlorophyll abundance and energy absorption^52^, and as a result the NDVI is correlated with LAI and FPAR^53^. However, the stratification of canopies in the Amazon into under- and overstory make translating changes in NDVI into changes in states (e.g. LAI) or fluxes (e.g., GPP) challenging^48^. In 2018, we found significant seasonal change in GOES ABI NDVI at four flux tower sites out of seven sites examined from the Amazon evergreen forest (Supplementary Figs. 3 and 4 and Supplementary Table 2). Our basin-wide analysis also showed that GOES ABI could not detect any seasonal changes in the remaining 15% of the Amazon evergreen forest zone, which may reflect the saturation of NDVI at high values of LAI and/or residual atmospheric contamination. For example, ground observations of LAI at Km34 showed clear seasonality from 5.5 in July and August to 6.5 in November and December^26^, while the GOES ABI did not show statistically significant seasonality in 16-day MVC NDVI (Fig. 2a and Supplementary Fig. 3a). All flux data from the Amazon evergreen forest show seasonality in NEE and GPP corresponding to precipitation seasonality (Supplementary Figs. 3 and 4). However, three out of seven sites did not show significant seasonality in the GOES ABI NDVI in 2018 (Supplementary Fig. 3a, e, f). Those results reveal that there remain some limitations in capturing the seasonality of biophysical parameters in dense forest canopies even using high-frequency observations of visible – shortwave radiation data. The synergetic use of space lidar, which can observe the surface even under cloud cover, could help to observe the seasonality of the Amazon evergreen forest over the region where the ABI could not capture seasonality. The spaceborne lidars also can contribute to understanding seasonal changes in forest structure under the canopy (e.g. ref. ^54^).

Regarding the issue of the Bidirectional Reflectance Distribution Function (BRDF), the small seasonal change in Amazon canopy greenness raises the possibility that it is an artifact of sun-sensor geometry^12,14^. The sun angle of an ABI observation changes from hour to hour unlike those from sun-synchronous satellite sensors, while the viewing angle on a per-pixel basis is constant–for example GOES-16 is located at 75.2°W in the northwest portion of the Amazon basin. Model studies showed that the NDVI of the Amazon has very small sensitivity to sun-sensor geometry compared to EVI and a correction for geometry did not affect the seasonal change^12,55^. As we only used the data between 9 a.m. and 3 p.m., the BRDF effect on NDVI is negligible in this study. There should be some remaining BRDF effects on TOA NDVI, but our conclusions cannot be explained by BRDF effects because the regional dependency of NDVI of Fig. 6 and Supplementary Fig. 2 is unrelated to the change in solar zenith angle.

A similar methodology to capture seasonal change was well validated in temperate forests using Himawari-8 Advanced Himawari Imager (AHI) data^56^. The same type of analysis used in this study can be applied to other cloudy tropical regions, i.e. Southeast Asia and central Africa. Sensors similar to ABI but with different spatial coverage, such as the Himawari-8 AHI, FengYun-4 Advanced Geosynchronous Radiation Imager (AGRI), and Meteosat Third Generation (MTG) Flexible Combined Imager (FCI), are able to enhance the long-term study of tropical evergreen forest. Correspondingly, the effort to combine new geostationary satellite sensors for analyses at the global scale will be useful for analysis of the entire tropical region.

We have shown evidence that GOES ABI data are required to extend the discussion of seasonal and long-term trends in the Amazon tropical forests. The high-frequency feature of GOES ABI allows analyses to minimize cloud contamination from Amazon evergreen forest images effectively. In addition, we conclude that Amazon basin analyses that only use sun-synchronous satellite sensors are not trustworthy for revealing seasonal change in greenness due to the residual cloud and aerosol contamination.

By taking advantage of high-frequency observations, the GOES ABI data revealed that seasonality occurs in 85% of Amazon evergreen forests which the sun-synchronous sensors were not able to detect. We also found that ABI facilitates more detailed mapping of Amazon evergreen forest seasonality, whereas MODIS or lidar images could not analyze such regional and high-temporal seasonality. Algorithms to correct for residual aerosol and BRDF for ABI are in progress to generate surface reflectance as GOES products. These correction algorithms will be able to contribute more precise analysis of seasonal change or long-term trends in Amazon evergreen forests in the future.

## Methods

### Two study sites (Km34 and RJA)

To scrutinize hourly and seasonal change in NDVI of the Amazon basin, we picked two well-studied sites, Reserva Cuieiras near Manaus (designated “Km34,” 2.61°S, 60.21°W)^32^, and Reserva Jaru (designated “RJA,” 10.08°S, 61.93°W)^37^. The land cover at Km34 is tropical evergreen broadleaf forest with mean annual precipitation of above 2500 mm/year. It has a weak dry season from approximately June to September. Most of rainfall occurs between 11 a.m. and 5 p.m. local time^32^. RJA is the same terra firme forest, but has a strong dry season from June to September. The landcover of RJA can be deemed tropical semideciduous forest as a subclass of tropical evergreen forest^57^. We extracted the 15-min NDVI time series from the corresponding ABI pixel in the 2018 NDVI data to represent seasonal reflectance variations of Amazon evergreen forests.

### GOES-16 ABI data

We calculated TOA NDVI from band 2 and band 3 TOA reflectances of GOES-16 ABI in 2018 and 2019. The TOA reflectance is one of the GOES-16 ABI products generated by the Geostationary-NASA Earth Exchange (GeoNEX) project^58^. Band 2 has a 0.64 μm central wavelength and a 500 m spatial resolution at nadir, while band 3 has a 0.86 μm central wavelength and a 1 km spatial resolution. We averaged the 500 m band 2 data for every 2 × 2 pixel to create a compatible 1 km resolution to use with the native 1 km band 3. We geocorrected the 1-km band 2 and band 3 data using the original 500-m band 2 data to match the coastlines of North and South America. Reflectance was calculated by dividing the radiance data by the cosine of the solar zenith angle. We then calculated the 1-km TOA NDVI from 1-km band 2 and band 3 TOA reflectance. Details of the process can be found in ref. ^58^.

We used TOA NDVI instead of surface NDVI. Despite ongoing efforts to correct atmospheric and angular (e.g., BRDF) effects from the ABI data^58^, additional uncertainties may be introduced in these corrections, which are still under evaluation. Therefore, we used a straightforward TOA NDVI in this analysis and adopted robust techniques to address the cloud contamination issue.

### MODIS datasets

The collection 6 MODIS NDVI products, MOD13A2 and MYD13A2, contain 16-day composites of 1 km NDVI from the Terra and Aqua platforms, respectively^48^. The MODIS NDVI products are calculated from the surface reflectance, not TOA reflectance, and corrected for the atmosphere and BRDF. The pixel reliability data in MOD13A2 and MYD13A2 were also extracted. A reliability code of 0 means, “Good data—use with confidence”, while 1 means, “Marginal data – Useful, but look at other QA information.” The overpass time of Terra and Aqua are approximately 10:30 a.m. and 1:30 p.m. local time, respectively. The precise overpass times of Terra and Aqua were obtained from MOD09GA and MYD09GA surface reflectance datasets, which contain the daily overpass information as metadata. As we did with ABI data, we extracted the time series of the MODIS product pixel data at Km34 and RJA in 2018.

In addition, we compared the MODIS MAIAC 1-km reflectance product, MCD19A1 version 6^59^, with ABI data. Because the MAIAC algorithm can provide more cloud-cleared data in the Amazon basin^31^, several studies have used MCD19A1 to calculate VIs for analysis of Amazon evergreen forest^44,60^. The MCD19A1 contains MODIS reflectance bands and QA flags including a snow flag. Besides the cloud screening, MAIAC data are characterized by atmospheric correction and BRDF correction applied to MODIS reflectance. We extracted both NDVI and EVI information for MODIS MAIAC products. The EVI was designed to correct the residual aerosol effect by utilizing the aerosol sensitivity of the MODIS blue band^48^, and has more sensitivity to dense canopy structure than NDVI. Therefore, EVI has been frequently used for monitoring the seasonal change in Amazon forest greenness.

To exclude other than evergreen forest landcover, we identified the pixels containing evergreen forest in the South America by using the MCD12Q1 land cover product^61^ (see Supplementary Fig. 1).

### Monthly precipitation data

We used the 0.25-degree Precipitation Estimation from Remotely Sensed Information using Artificial Neural Networks (PERSIANN) monthly precipitation data^62^ to show the seasonal pattern of precipitation in 2018. The time series was extracted from the pixel containing the target point.

### AErosol RObotic NETwork (AERONET) data

We used spectral AOD data from the Level 2 AERONET database Version 3^63^. Three sites in the target area had sufficient AOD data in 2018 (ATTO 2.14°S, 59.00°W, Alta Floresta Tower 9.87°S, 56.10°W, and Ji Parana SE Tower 10.93°S, 61.85°W) (see the location in Supplementary Fig. 1). AOD were provided every 15 min under clear sky conditions. We used the AOD at 500 nm. To identify the AOD at the time of MVC NDVI, we searched the AOD observations at the time between plus and minus 7.5 minutes of satellite sensor observed time.

### Maximum value composite (MVC)

The MVC was developed to minimize atmospheric effects on NDVI^33^. The MVC finds the highest NDVI in the defined time period (e.g. 16-day or month) and assumes that the highest NDVI is the closest value to the NDVI without atmospheric effects.

### Statistical test to identify the seasonality of the evergreen forest

Unlike MODIS observation data, GOES 16-day MVC can provide sufficiently clear day NDVI in the wet seasons to apply statistical significance tests for the seasonality in NDVI of Amazon evergreen forest. First, we defined the high-NDVI season as the consecutive three months that have the highest mean NDVI, and the low-NDVI season as the consecutive three months that have the lowest mean NDVI. The consecutive three-month period should have six 16-day MVC NDVIs. Then, we used the one-sided two-sample *t* test to determine the significance of the difference in NDVIs between the high-NDVI season and the low-NDVI season using the Python SciPy function “ttest_ind”^64^. The null hypothesis of the test was that there was no difference between the set of NDVIs of the two seasons, and we rejected the null hypothesis when the *p*-value was less than 0.05. For every pixel classified as evergreen forest, we plotted the first month of the high-NDVI season (Fig. 5g) and the low-NDVI season (Fig. 5h) when the NDVI in the two seasons were statistically significant.

### Reporting summary

Further information on research design is available in the Nature Research Reporting Summary linked to this article.

## Supplementary information

Supplementary Information

Reporting Summary

## Data Availability

All GOES ABI data used in this study are publicly available through the NASA GeoNEX website: https://www.nasa.gov/geonex/dataproducts. All MODIS data used in this study are publicly available through NASA’s Land Processes Distributed Active Archive Center (LP DAAC: https://lpdaac.usgs.gov. Tower data are publicly available through the Large-Scale Biosphere-Atmosphere Experiment in Amazonia, LBA-ECO: 10.3334/ORNLDAAC/1174 and FLUXNET 2015: https://fluxnet.org/data/fluxnet2015-dataset/.

## Code availability

Python code to handle GOES ABI TOA products at NASA GeoNEX is available at 10.5281/zenodo.4318065.
