# Supplementary material for: New generation geostationary satellite observations support seasonality in greenness of the Amazon evergreen forests
Source: Nat Commun. 2021 Jan 29;12:684. doi: 10.1038/s41467-021-20994-y (PMC7846599; doi:10.1038/s41467-021-20994-y)
Supplement: Supplementary file 1 — Supplementary Information [file 41467_2021_20994_MOESM1_ESM.docx]

**Supplementary Information**

New generation geostationary satellite observations support seasonality in greenness of the Amazon evergreen forests

Hirofumi Hashimoto, Weile Wang, Jennifer L. Dungan, Shuang Li, Andrew R. Michaelis, Hideaki Takenaka, Atsushi Higuchi, Ranga B. Myneni, and Ramakrishna R. Nemani

Including:

**Supplementary Fig. 1.**

**Supplementary Fig. 2.**

**Supplementary Fig. 3.**

**Supplementary Fig. 4.**

**Supplementary Table 1.**

**Supplementary Table 2.**

**Supplementary References**

**Supplementary Fig. 1.**


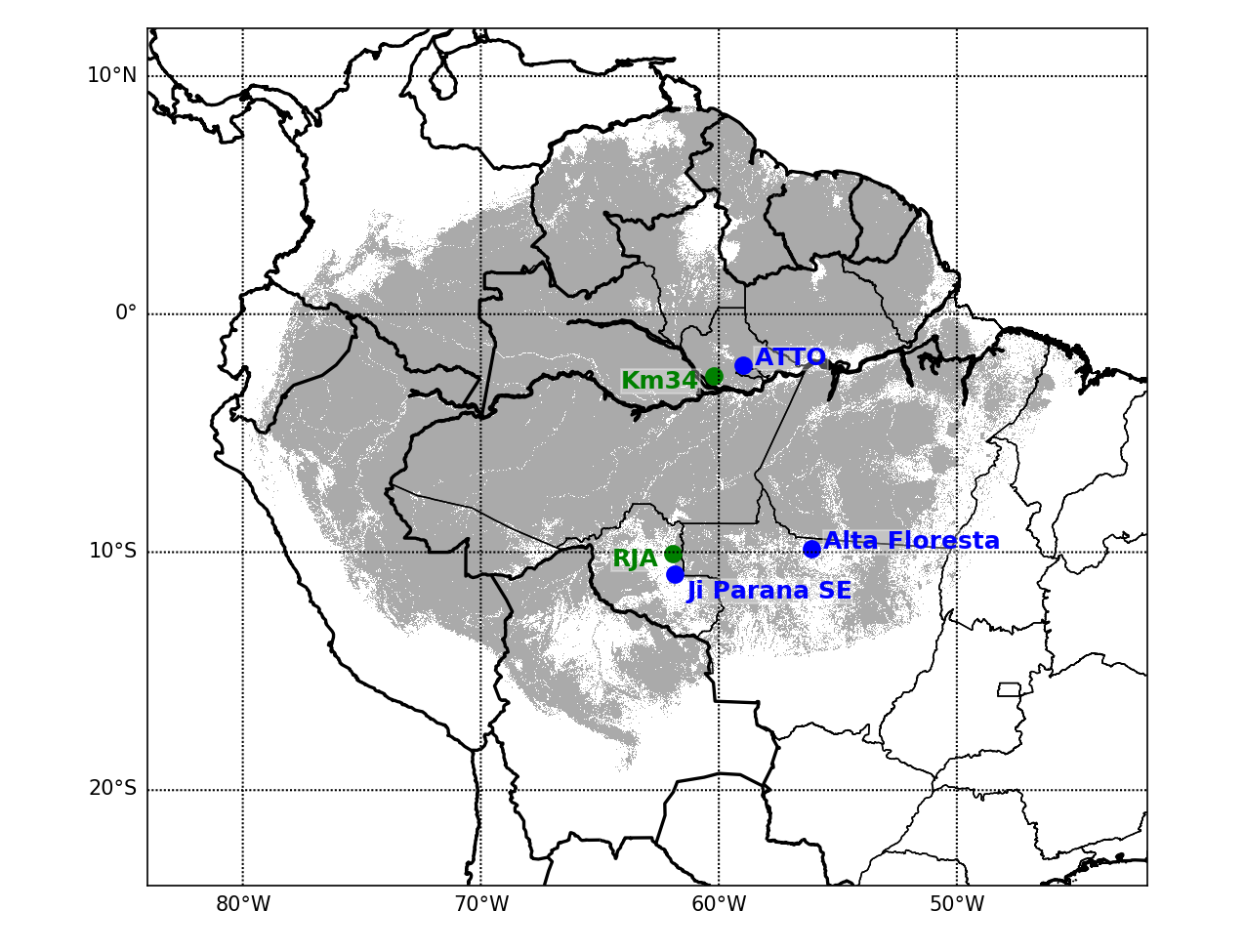


**Supplementary Fig. 1 | Target area and the location of study sites.** The green dots are flux observation sites (Km34 and RJA). The blue dots are Aerosol Robotic Network (AERONET) sites (ATTO, Alta Floresta, and Ji Parana SE). The target area is shown in gray.

**Supplementary Fig. 2.**

**
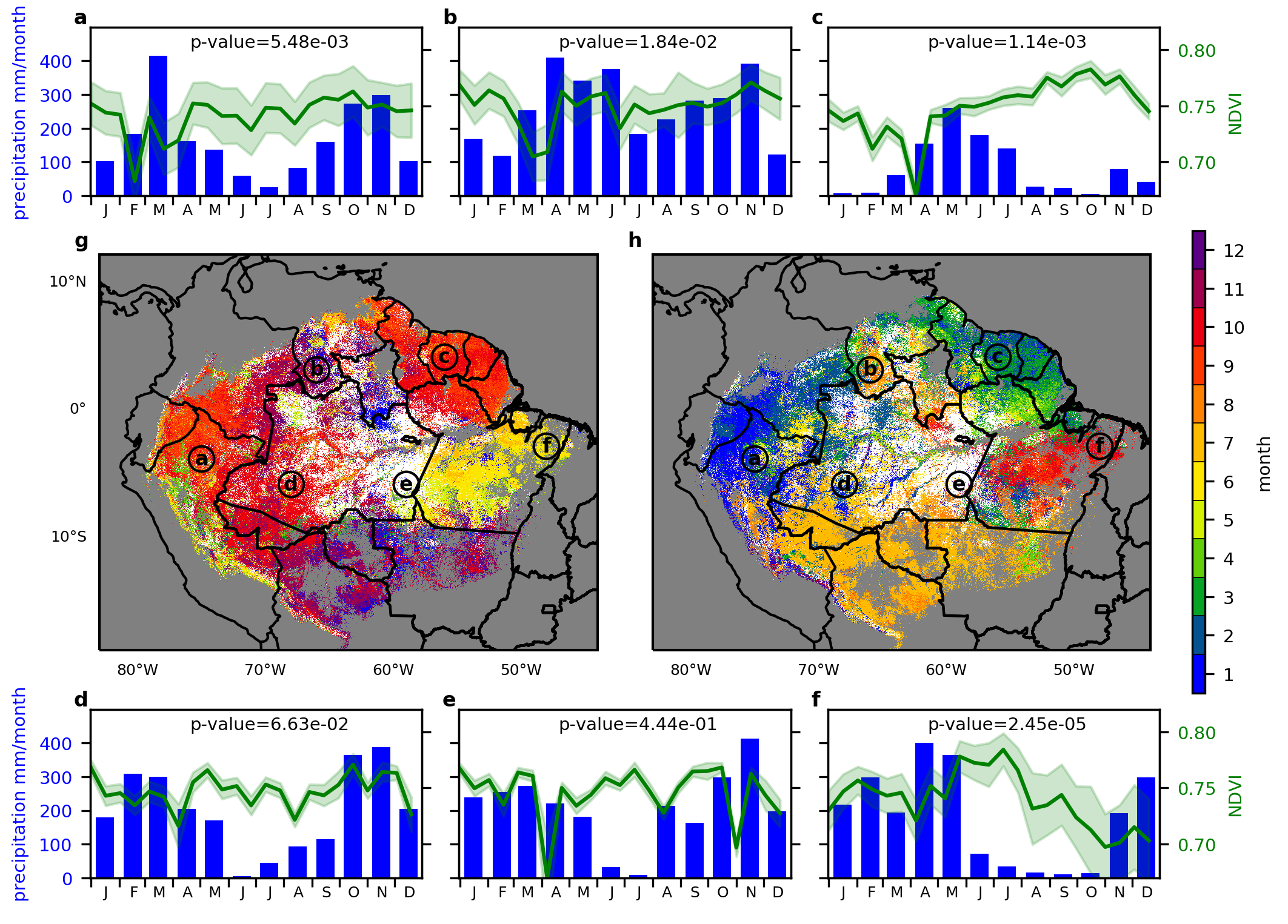
**

**Supplementary Fig. 2 | Same figure as Fig. 6 except for 2019 analysis.** Panels **a** to **f** show example time series of 16-day MVC NDVI corresponding to the six locations (a-f) indicated on the map g and h. The green line and envelopes are the mean and standard deviation, respectively, of NDVI taken from the 11 x 11 pixel region surrounding each location. The blue bars show monthly precipitation at each location. The two maps show the first month of the consecutive three months in which daily MVC NDVI reaches its (g) highest and (h) lowest values during 2019. Pixels where the high-to-low NDVI differences are statistically insignificant (p>0.05) by the two-sample t tests are masked out and shown in white.

**Supplementary Fig. 3.**

**
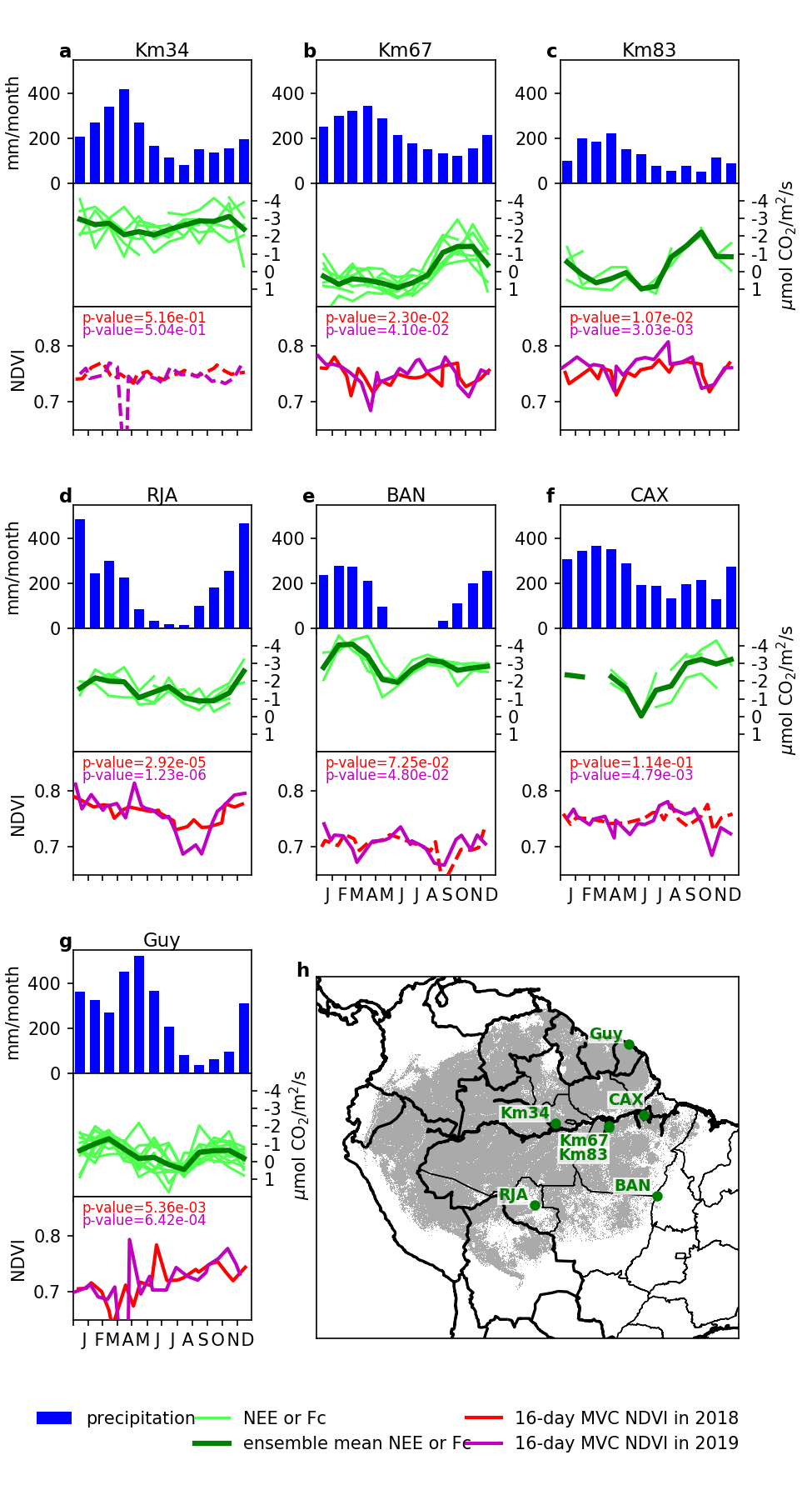
**

**Supplementary Fig. 3 | Comparison of seasonality among precipitation, carbon flux, and GOES ABI NDVI at seven flux tower sites.** (**a-g**) The dark blue bars are mean monthly precipitation (mm/year) measured at flux tower sites. The light green lines are monthly mean Net Ecosystem Exchange (NEE) or CO_2_ flux at the top of the canopy (Fc) of available years measured at flux towers. The thick green lines are ensemble means of the light green lines. NEE where its quality flag was higher than 0.5 is shown. 16-day MVC ABI NDVI in 2018 and 2019 are shown in red and magenta, respectively. The NDVI lines are solid when the seasonality of the 16-day MVC ABI NDVI is significant (p<0.05) using the one-sided two-sample t test (broken line when insignificant). ¥(**h**) The green dots show the location of the seven flux observation sites. The target area is shown in gray. See the summary descriptions of each site in Supplementary Table 2.

**Supplementary Fig. 4.**

**
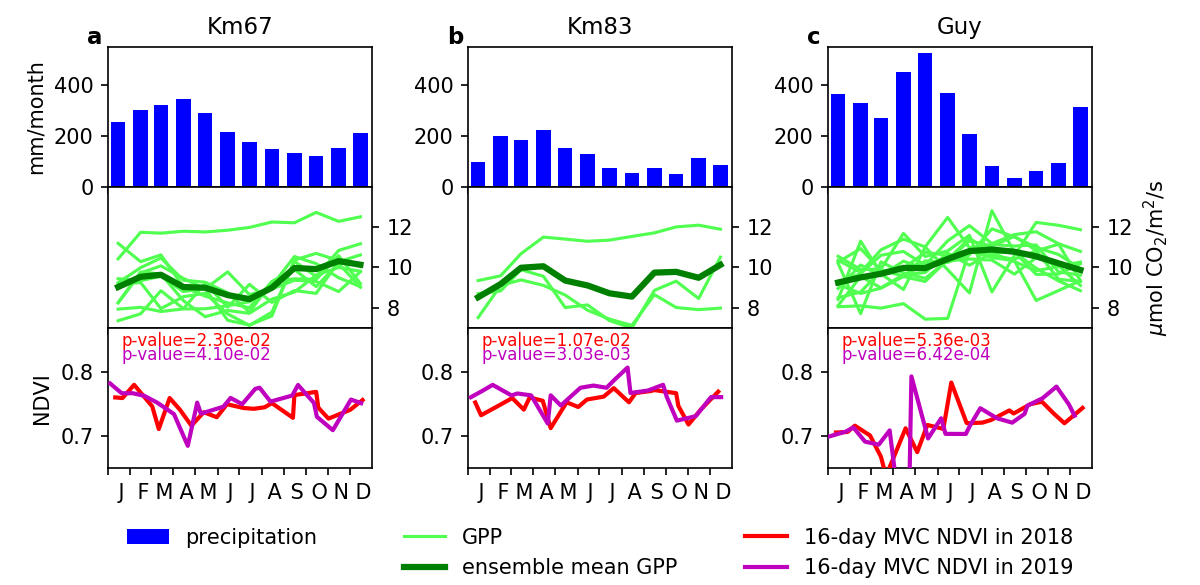
**

**Supplementary Fig. 4 | Comparison of seasonality among precipitation, carbon flux, and GOES ABI NDVI at three flux tower sites.** (**a-c**) The dark blue bars are mean monthly precipitation (mm/year) measured at flux tower sites. The light green lines are monthly mean Gross Primary Production (GPP) of available years measured at flux towers. The thick green lines are ensemble means of the light green lines. GPP where its quality flag was higher than 0.5 is shown. 16-day MVC ABI NDVI and p-value in 2018 and 2019 are shown in red and magenta, respectively. P-value is calculated using the one-sided two-sample t test. See the summary descriptions of each site in Supplementary Table 2.

**Supplementary Table 1 | Mean number of clear-sky observations per month in 2018.** The numbers of clear-sky observations per month (Fig. 3) are averaged over all Amazon evergreen forest pixels for all months in 2018.

| Month | 9 a.m. - 3 p.m. every 15 minutes | 10:30 a.m.  Terra MODIS overpass time | 1:30 p.m. Aqua MODIS overpass time |
| --- | --- | --- | --- |
|  | (count/month) | (count/month) | (count/month) |
| Jan | 25.2 | 0.9 | 1.1 |
| Feb | 23.6 | 1.0 | 0.9 |
| Mar | 23.4 | 0.9 | 0.9 |
| Apr | 23.4 | 0.7 | 1.1 |
| May | 46.9 | 1.9 | 1.8 |
| Jun | 90.7 | 4.2 | 3.0 |
| Jul | 120.0 | 5.6 | 4.1 |
| Aug | 66.6 | 2.8 | 2.5 |
| Sep | 58.7 | 2.5 | 2.1 |
| Oct | 37.4 | 1.4 | 1.5 |
| Nov | 21.2 | 0.6 | 0.9 |
| Dec | 23.1 | 0.8 | 1.0 |
| mean | 46.7 | 1.9 | 1.7 |

**Supplementary Table 2 | Summary description of flux tower sites used in Supplementary Figure 3 and 4.** Data sources for precipitation, Net Ecosystem Exchange (NEE), and CO_2_ flux at the top of the canopy (Fc) were LBA-ECO (Saleska et al., 2013) and monthly FLUXNET2015 (Pastorello et al., 2020). Due to data availability, we presented NEE for FLUXNET2015 and Fc for LBA-ECO in Supplementary Fig. 3. While several NEE and GPP statistics were available in FLUXNET2015, we chose NEE_VUT_REF and GPP_NT_VUT_REF in this analysis. The mean annual precipitation was the average of years when NEE or CO_2_ flux at the top of Fc is available.

| site ID | site name | latitude (º) | longitude (º) | vegetation classification | Mean annual precipitation (mm/year) | source of Fc or NEE | reference |
| --- | --- | --- | --- | --- | --- | --- | --- |
| Km34 | Manaus | -2.609 | -60.209 | Evergreen Broadleaf Forest | 2517 | LBA-ECO | Araújo et al. (2002) |
| Km67 | Santarem | -2.857 | -54.959 | Evergreen Broadleaf Forest (Primary Forest) | 2682 | FLUXNET2015 | Saleska et al. (2003) |
| Km83 | Santarem | -3.018 | -54.971 | Evergreen Broadleaf Forest (Logged Forest) | 1475 | FLUXNET2015 | Saleska et al. (2003) |
| RJA | Reserva Jaru | -10.078 | -61.933 | Evergreen Broadleaf Forest | 2420 | LBA-ECO | Kruijt et al. (2004) |
| BAN | Ilha do Bananal | -50.159 | -9.824 | seasonally flooded transitional forest | 1704 | LBA-ECO | Borma et al. (2009) |
| CAX | Caxiuana National Forest | -1.748 | -51.454 | Evergreen Broadleaf Forest | 2998 | LBA-ECO | Carswell et al. (2002) |
| Guy | Guyaflux | 5.279 | -52.925 | Evergreen Broadleaf Forest | 3110 | FLUXNET2015 | Bonal et al. (2008) |
|  |  |  |  |  |  |  |  |

**Supplementary References**

Araújo, A. C., *et al.* Comparative measurements of carbon dioxide fluxes from two nearby towers in a central Amazonian rainforest: The Manaus LBA site. *Journal of Geophysical Research*, *107*(D20), 8090 (2002). https://doi.org/10.1029/2001JD000676

Bonal, D., *et al.* Impact of severe dry season on net ecosystem exchange in the Neotropical rainforest of French Guiana. *Global Change Biology*, *14*(8), 1917–1933 (2008). https://doi.org/10.1111/j.1365-2486.2008.01610.x

Borma, L. S., *et al*. Atmosphere and hydrological controls of the evapotranspiration over a floodplain forest in the Bananal Island region, Amazonia. *Journal of Geophysical Research: Biogeosciences*, *114*(1), G01003 (2009). https://doi.org/10.1029/2007JG000641

Carswell, F. E., *et al.* Seasonality in CO_2_ and H2O flux at an eastern Amazonian rain forest. *Journal of Geophysical Research: Atmospheres*, *107*(20), LBA 43-1-LBA 43-16 (2002). https://doi.org/10.1029/2000JD000284

Kruijt, B., *et al.* The robustness of eddy correlation fluxes for Amazon rain forest conditions. *Ecological Applications*, *14*(4 SUPPL.), 101–113 (2004). https://doi.org/10.1890/02-6004

Pastorello, G., *et al.* The FLUXNET2015 dataset and the ONEFlux processing pipeline for eddy covariance data. *Scientific Data*, *7*(1), 225 (2020). https://doi.org/10.1038/s41597-020-0534-3

Saleska, S. R., *et al. LBA-ECO CD-32 Flux Tower Network Data Compilation, Brazilian Amazon: 1999-2006.* 1999–2006 (2013). https://doi.org/10.3334/ORNLDAAC/1174

Saleska, S. R., *et al.* Carbon in Amazon Forests: Unexpected Seasonal Fluxes and Disturbance-Induced Losses. *Science*, *302*(5650), 1554–1557 (2003). https://doi.org/10.1126/science.
