# Supplementary material for: New generation geostationary satellite observations support seasonality in greenness of the Amazon evergreen forests
Source: Nat Commun. 2021 Jan 29;12:684. doi: 10.1038/s41467-021-20994-y (PMC7846599; doi:10.1038/s41467-021-20994-y)
Supplement: Supplementary file 2 — Reporting Summary [file 41467_2021_20994_MOESM2_ESM.pdf]

nature research

Corresponding author(s): Hirofumi Hashimoto
Last updated by author(s): Dec 14, 2020

Reporting Summary

Nature Research wishes to improve the reproducibility of the work that we publish. This form provides structure for consistency and transparency in reporting. For further information on Nature Research policies, see our Editorial Policies and the Editorial Policy Checklist.

Statistics

For all statistical analyses, confirm that the following items are present in the figure legend, table legend, main text, or Methods section.

n/a	Confirmed	
☐	☒	The exact sample size (n) for each experimental group/condition, given as a discrete number and unit of measurement
☒	☐	A statement on whether measurements were taken from distinct samples or whether the same sample was measured repeatedly
☐	☒	The statistical test(s) used AND whether they are one- or two-sided<br>*Only common tests should be described solely by name; describe more complex techniques in the Methods section.*
☒	☐	A description of all covariates tested
☐	☒	A description of any assumptions or corrections, such as tests of normality and adjustment for multiple comparisons
☐	☒	A full description of the statistical parameters including central tendency (e.g. means) or other basic estimates (e.g. regression coefficient) AND variation (e.g. standard deviation) or associated estimates of uncertainty (e.g. confidence intervals)
☐	☒	For null hypothesis testing, the test statistic (e.g. F, t, r) with confidence intervals, effect sizes, degrees of freedom and P value noted<br>*Give P values as exact values whenever suitable.*
☒	☐	For Bayesian analysis, information on the choice of priors and Markov chain Monte Carlo settings
☒	☐	For hierarchical and complex designs, identification of the appropriate level for tests and full reporting of outcomes
☒	☐	Estimates of effect sizes (e.g. Cohen's d, Pearson's r), indicating how they were calculated

Our web collection on statistics for biologists contains articles on many of the points above.

Software and code

Policy information about availability of computer code

Data collection	No software was used
Data analysis	Python 2.7.15 and Scipy 1.1.0. The Python code handling GOES ABI TOA products at NASA GeoNEX is available at https://github.com/GeoNEX-Community-Tools/GeoNEX-Science-Tutorials

For manuscripts utilizing custom algorithms or software that are central to the research but not yet described in published literature, software must be made available to editors and reviewers. We strongly encourage code deposition in a community repository (e.g. GitHub). See the Nature Research guidelines for submitting code & software for further information.

Data

Policy information about availability of data

All manuscripts must include a data availability statement. This statement should provide the following information, where applicable:
- Accession codes, unique identifiers, or web links for publicly available datasets
- A list of figures that have associated raw data
- A description of any restrictions on data availability

All GOES ABI data used in this study are publicly available through the NASA GeoNEX website: https://www.nasa.gov/geonex/dataproducts. All MODIS data used in this study are publicly available through NASA's Land Processes Distributed Active Archive Center (LP DAAC: https://lpdaac.usgs.gov). Tower data are publicly available through the Large-Scale Biosphere-Atmosphere Experiment in Amazonia, LBA-ECO: https://doi.org/10.3334/ORNLDAAC/1174 and FLUXNET: https://fluxnet.org/data/fluxnet2015-dataset/.

Field-specific reporting

Please select the one below that is the best fit for your research. If you are not sure, read the appropriate sections before making your selection.

☐ Life sciences ☐ Behavioural & social sciences ☒ Ecological, evolutionary & environmental sciences

For a reference copy of the document with all sections, see nature.com/documents/nr-reporting-summary-flat.pdf

Ecological, evolutionary & environmental sciences study design

All studies must disclose on these points even when the disclosure is negative.

Study description	We analyzed GOES-16 Advanced Baseline Imager (ABI) data to examine whether the ABI supports the notion of seasonality in the greenness of the Amazon evergreen forest. Evidence from ABI allows us to peek through ephemeral clear skies over the land surface of the Amazon basin and show that on average, ABI had approximately 25 times more observations than the previously used polar-orbiting sensor, the Moderate Resolution Imaging Spectroradiometer (MODIS). The ABI data revealed seasonality in 85% of Amazon evergreen forest pixels with fine spatiotemporal resolution. Our findings confirm the existence of canopy seasonality, previously only barely detectable with MODIS.
Research sample	We used the existing dataset GOES-16 ABI full disk data. The dataset was produced by NOAA and the description can be found in https://www.goes-r.gov/users/docs/PUG-L1b-vol3.pdf in their website. We converted the full-disk images to a geographic coordinate system. https://www.nasa.gov/geonex/dataproducts
Sampling strategy	We used all the available data every 15 minutes in 2018 and 2019.
Data collection	The new sensor ABI on GOES-16 measures the reflectance and radiometric temperature at the top of the atmosphere. ABI's full disk scan covers the entire Amazon basin. In 2018, the ABI default mode of scanning was Mode 3, which performs full disk scans every 15 minutes. The frequency of ABI full-disk observations became 10 minutes after April 2019. NOAA received the signals from GOES-16 and formatted the data to NetCDF file format. We collected all the available full-disk data in 2018 and 2019.
Timing and spatial scale	We collected the full disk image of the GOES ABI data from 2018 January 1st 00 UTC to 2019 December 31st 24 UTC every 10 or 15 minutes. The full-disk image covers North and South America; our study is limited to Amazon ever green forest.
Data exclusions	Maximum value compositing was used to filter MODIS and ABI data that are presumed to represent cloud-contaminated conditions.
Reproducibility	This is an Earth observation study. Methods initially applied to data from 2018 were subsequently applied to data from 2019 with the same conclusions drawn. Given the data and code are publicly available, independent researchers have the ability to reproduce the methods and results.
Randomization	Randomization is not relevant because we used all the available data in 2018 and 2019 for every pixel.
Blinding	Data were derived from satellite sensor measurements that are publicly available.

Did the study involve field work? ☐ Yes ☒ No

Reporting for specific materials, systems and methods

We require information from authors about some types of materials, experimental systems and methods used in many studies. Here, indicate whether each material, system or method listed is relevant to your study. If you are not sure if a list item applies to your research, read the appropriate section before selecting a response.

Materials & experimental systems

n/a	Involved in the study	
☒	☐	Antibodies
☒	☐	Eukaryotic cell lines
☒	☐	Palaeontology and archaeology
☒	☐	Animals and other organisms
☒	☐	Human research participants
☒	☐	Clinical data
☒	☐	Dual use research of concern

Methods

n/a	Involved in the study	
☒	☐	ChIP-seq
☒	☐	Flow cytometry
☒	☐	MRI-based neuroimaging
